# Pasteurella multocida Infection of an Aortic Endograft: A Rare Zoonotic Complication

**DOI:** 10.7759/cureus.111052

**Published:** 2026-06-17

**Authors:** August Anderson, John M West, Andrew W Chao

**Affiliations:** 1 Department of Medicine, Medical College of Georgia, Augusta University, Augusta, USA

**Keywords:** aortic graft infection, endovascular abdominal aortic aneurysm repair, infectious aortitis, pasteurella multocida, zoonotic infections

## Abstract

*Pasteurella multocida* is a zoonotic, encapsulated, and gram-negative coccobacillus frequently implicated in soft tissue infections following animal bites, particularly dogs and cats. Invasive infections, including bacteremia and endovascular involvement, are rare and typically occur in immunocompromised or elderly individuals. Vascular graft infections due to *P. multocida* are rare, with limited cases reported in the literature.

We report the case of an 87-year-old man with a history of abdominal aortic aneurysm repair who presented with acute confusion, weakness, and dyspnea. Initial workup revealed leukocytosis, right lower lobe consolidation, and bacteremia with *P. multocida*. Further history uncovered a recent dog bite to the hand. Given the patient’s vascular history, CT angiography was obtained revealing inflammatory changes surrounding the aortic graft, concerning for infectious aortitis. Due to the patient’s advanced age and comorbidities, surgical intervention was deferred. He was treated with a four-week course of intravenous ceftriaxone followed by chronic oral doxycycline. Serial imaging with CT angiography over 18 months demonstrated clinical and radiographic stability, and suppressive antibiotic therapy was eventually discontinued without recurrence.

This case highlights a rare but serious complication of *P. multocida *bacteremia involving vascular graft infection. In select patients with high surgical risk, long-term suppressive antibiotic therapy may offer a viable alternative to surgical intervention. Clinicians should maintain a high index of suspicion for endovascular infection in bacteremic patients with prosthetic vascular materials and recent animal exposure, even in the absence of overt localizing symptoms.

## Introduction

*Pasteurella multocida* is an encapsulated, gram-negative zoonotic organism widely carried by domestic and wild animals, with human infections most commonly arising after dog or cat bites [1]. Although typically limited to soft tissue involvement, *P. multocida* is capable of causing invasive disease, particularly in elderly or immunocompromised hosts. Reported manifestations include bacteremia, respiratory tract infections, and endovascular involvement; however, vascular graft infections caused by *P. multocida* are rare [1]. Management of infected aortic grafts is challenging and generally relies on prompt diagnosis, source control, and prolonged antimicrobial therapy. When operative management is precluded by patient-specific factors, clinicians face difficult decisions regarding long-term suppressive treatment and monitoring. Here, we present a case of *P. multocida* bacteremia complicated by probable infection of an abdominal aortic endograft, managed successfully with extended antibiotic therapy in lieu of surgery.

## Case presentation

An 87-year-old male with a past medical history of chronic obstructive pulmonary disease (COPD), hyperlipidemia, gastroesophageal reflux disease, abdominal aortic aneurysm (AAA) with endovascular aneurysm repair six years prior, migraines, and status post inguinal hernia repair presented to the emergency department with confusion, generalized weakness, and inability to ambulate for two days.

His wife reported that he had become progressively weak and confused over the previous day, with poor oral intake. She denied any history of trauma or recent falls. The patient had been recently diagnosed with pneumonia and was prescribed supplemental oxygen at home as well as a course of antibiotics. On presentation, he was on 4 L/min of oxygen via a nasal cannula. He endorsed feeling weak but denied other symptoms aside from a chronic cough and dyspnea, which he attributed to his underlying COPD. He specifically denied fever, chest pain, gastrointestinal symptoms, dysuria, or any focal neurological deficits leading up to this illness.

Initial evaluation revealed leukocytosis to 17.3 × 10⁹/L (reference range: 4.5-11.0 × 10⁹/L), tachypnea with a respiratory rate of 27 (reference range: 12-20), and a right lower lobe consolidation on chest radiograph (see Figure 1). Given concern for sepsis versus a COPD exacerbation, he was empirically treated with intravenous (IV) antibiotics, receiving a single dose of ceftriaxone 1000 mg and doxycycline 100 mg. He was admitted and transitioned to cefepime 2000 mg every eight hours and vancomycin dosed by pharmacy. A non-contrast head CT was negative, and urinalysis was not indicative of infection.

**Figure 1:**
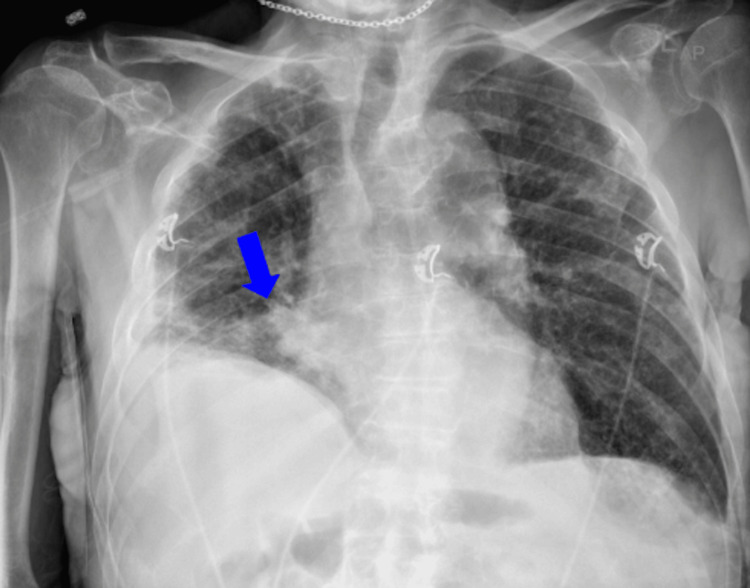
Chest radiograph The figure demonstrates the initial anteroposterior (AP) chest radiograph showing evidence of right lower lobar consolidation (blue arrow).

Upon empiric treatment, the patient had improvement in his leukocytosis and tachypnea. His mental status and weakness also gradually improved. On hospital day three, admission blood cultures grew *P. multocida*, along with anaerobic gram-negative cocci in clusters. The infectious diseases team was consulted for evaluation and discharge recommendations. Upon further history taking, the patient’s wife recalled that he had recently been bitten on the right hand while feeding one of their dogs. Specifically, he enjoys feeding his dogs out of his hands and periodically will sustain superficial bites, with the last being approximately one to two weeks before presentation. The patient himself, still encephalopathic, did not recall the event. There was no cellulitis or abscess at his most recent bite.

Given his history of AAA repair and current bacteremia, CT angiography (CTA) of the abdomen with contrast was ordered and obtained on hospital day four. Imaging demonstrated aortic wall thickening and surrounding inflammatory changes in the distal abdominal aorta just proximal to the bifurcation, concerning for infectious aortitis (see Figure 2). Vascular surgery was consulted and recommended no acute surgical intervention. Due to the patient’s advanced age and comorbidities, surgery was felt to carry more risk than benefit, so medical treatment alone was pursued.

**Figure 2:**
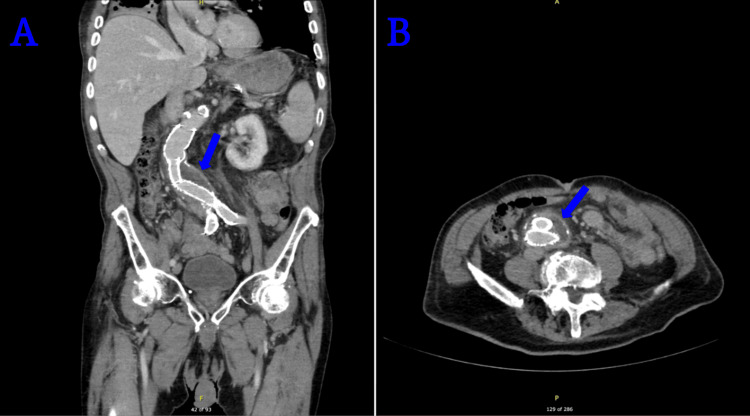
Computed tomography angiography of the abdomen and pelvis Coronal (A) and axial (B) slices demonstrating aortic wall thickening with surrounding inflammatory changes of the distal abdominal aorta concerning for infectious aortitis (blue arrows).

Repeat blood cultures obtained on hospital day two and hospital day three demonstrated no growth. A peripherally inserted central catheter was placed, and the patient was transitioned to IV ceftriaxone to complete a planned four-week course of initial therapy. A transthoracic echocardiogram (TTE) showed mildly reduced right ventricular function with trace aortic, mitral, and tricuspid regurgitation; although no evidence of vegetations was seen, valve visualization was limited, and endocarditis could not be definitively ruled out. Further workup with a transesophageal echocardiogram (TEE) was pursued, but the patient declined this study. 

On hospital day eight, the patient was discharged to a subacute rehabilitation facility with outpatient follow-up. His confusion and weakness had largely resolved at the time of discharge. After completing the four-week IV ceftriaxone course, repeat blood cultures remained negative. Unfortunately, a subsequent CTA performed one month after finishing IV antibiotics showed increased infrarenal aortic sac wall enhancement and thickening, with persistent lymphadenopathy, concerning for ongoing infectious aortitis (see Figure 3). Vascular surgery again assessed the patient, but surgical intervention was deferred given the high risk of operative morbidity in the context of advanced age and comorbidities. Further IV antibiotic treatment was considered but not pursued due to insurance limitations and the patient's financial constraints. After shared decision-making with the patient and his wife, treatment with oral doxycycline 100 mg twice daily was begun.

**Figure 3:**
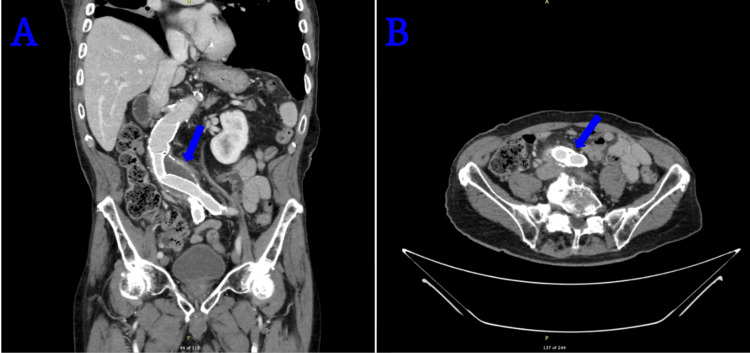
Repeat computed tomography angiography of the abdomen and pelvis Coronal (A) and axial (B) slices showing worsened aortic wall enhancement and thickening concerning for ongoing aortitis (blue arrows).

Serial CTAs over the next 18 months demonstrated at first stable, then improved inflammatory changes and interval reduction in aortic sac dimensions. Approximately 20 months after starting doxycycline, the patient was advised to discontinue doxycycline after sustained radiographic and clinical stability. A final follow-up CTA performed two months after stopping antibiotics showed no progression of disease.

## Discussion

*P. multocida* is a gram-negative coccobacillus commonly found in the oral flora of wild and domestic animals, particularly cats and dogs. In humans, it most frequently causes soft tissue infections following bites or scratches. However, more invasive manifestations have been reported, particularly in older or immunocompromised individuals, including bacteremia, endocarditis, osteomyelitis, and, rarely, vascular graft infections. A retrospective study of 482 *P. multocida* infections found that the majority occurred in elderly patients, with skin and soft tissue infections being the most common presentation, often following animal exposure [1]. *P. multocida* possesses several distinct virulence factors, including a polysaccharide capsule that prevents complement-mediated lysis and phagocytosis; lipopolysaccharide that triggers inflammatory responses; filamentous hemagglutinins and fimbriae that mediate adherence to host epithelial cells; and the Pasteurella multocida toxin, a potent mitogen that modulates host cell signaling by activating heterotrimeric G proteins, leading to cytoskeletal rearrangements and impaired immune function [2]. These factors collectively facilitate bacterial colonization, immune evasion, and tissue damage during infection. For mild or localized *P. multocida* infections, penicillin or oral amoxicillin-clavulanate remains the preferred treatment, though rare β-lactamase-producing resistant strains exist. More severe or systemic infections require aggressive parenteral therapy with agents such as ampicillin-sulbactam, piperacillin-tazobactam, carbapenems, or ceftriaxone often combined with anaerobic coverage [3].

Our patient presented uniquely with what was eventually found to be endovascular colonization of a graft from a prior AAA. *P. multocida* seldom establishes intravascular infections even during bacteremia, likely because its polysaccharide capsule, which is essential for avoiding phagocytosis, simultaneously inhibits biofilm formation on synthetic surfaces, reducing its ability to adhere to and colonize vascular prostheses [4]. Such cases have been reported in the literature but only rarely; Silberfein et al. reported an aortic endograft infection caused by *P. multocida* following a rabbit bite, with infection confirmed by cultures and managed with graft explantation [5]. Martin et al. described *P. multocida* causing a central venous catheter bloodstream infection, illustrating its capacity to infect intravascular devices without direct bite at the device site [6]. Teso et al. detailed a case of abdominal aortic endograft infection by *P. multocida* confirmed by imaging and blood cultures, managed with combined medical and surgical therapy [7].

Our case distinguishes itself from these in that surgical intervention was deferred due to our patient’s risk factors for intraoperative and postoperative morbidity and mortality. Long-term use of doxycycline was selected based on affordability and bioavailability and resulted in eventual control of the infection. Interpretation of the follow-up CTA obtained after completion of the course of IV ceftriaxone was challenging. Although the CTA demonstrated radiographic worsening, blood cultures had remained negative, and the patient remained clinically improved. Persistent radiographic abnormalities after treatment in this case may reflect ongoing inflammation rather than active microbial proliferation. Even so, additional IV antimicrobial therapy was preferred, but unfortunately, the patient encountered financial barriers which prevented this. 

An additional consideration in this case was the possibility of concomitant infective endocarditis. Although the TTE did not demonstrate valvular vegetations, valve visualization was limited. Further workup with a TEE was strongly encouraged, but this was declined by the patient. Consequently, endocarditis could not be definitively ruled out in this case. Given the absence of persistent bacteremia, the lack of definitive echocardiographic evidence of endocarditis on TTE, the lack of any overt signs or symptoms of endocarditis, and the patient's preference to forgo invasive testing, management focused instead on antimicrobial therapy for the aortic graft infection. The inability to definitively exclude endocarditis represents an inherent limitation of this case.

In this case, the absence of a feasible surgical source control decided the patient’s management strategy. Standard treatment for aortic graft infections typically prioritizes graft explantation with in situ or extra-anatomic reconstruction, as medical therapy alone carries a higher risk of persistent infection and late complications [8]. However, advanced age, significant comorbidities, limited functional reserve, and financial constraints made operative intervention and extended IV therapy impractical for this patient. These combined clinical, social, and economic factors necessitated a non-operative approach emphasizing prolonged suppressive therapy. Doxycycline was chosen for its oral bioavailability, tolerability, activity against *P. multocida*, and accessibility. Although data specifically evaluating doxycycline's penetration into infected aortic grafts are limited, its favorable bioavailability and activity against *P. multocida*, in addition to its practicality in this case, led to its selection. Nevertheless, evidence supporting doxycycline as the sole therapy for vascular graft infections remains limited, and its use in this case should be viewed as an individualized suppressive strategy rather than a replacement for standard management. Discontinuation of doxycycline therapy was guided by serial radiographic stability and eventual improvement in inflammatory changes surrounding the graft. Notably, the patient tolerated long-term doxycycline without adverse effects and experienced no recurrent bacteremia or hospitalizations attributable to *P. multocida*, supporting the viability of individualized, suppressive medical management in selected high-risk patients when definitive surgical source control is not an option.

## Conclusions

This report contributes a rare but educational example of aortic graft infection due to *P. multocida* managed non-operatively. It reinforces the principle that, in patients for whom graft explantation is not feasible due to several factors, including advanced age, comorbidity burden, and financial or social limitations, an extended course of suppressive antibiotic therapy can be safely considered, provided there is reliable monitoring and documented imaging stability. Clinicians should therefore maintain a low threshold for imaging evaluation of prosthetic vascular materials in the setting of *P. multocida* bacteremia and should engage in multidisciplinary decision-making regarding the trade-offs of surgical versus prolonged medical management.
